# Epigenome-wide Association Study Shows Differential DNA Methylation of *MDC1*, *KLF9*, and *CUTA* in Autoimmune Thyroid Disease

**DOI:** 10.1210/clinem/dgad659

**Published:** 2023-11-14

**Authors:** Nicole Lafontaine, Christopher J Shore, Purdey J Campbell, Benjamin H Mullin, Suzanne J Brown, Vijay Panicker, Frank Dudbridge, Thomas H Brix, Laszlo Hegedüs, Scott G Wilson, Jordana T Bell, John P Walsh

**Affiliations:** Department of Endocrinology & Diabetes, Sir Charles Gairdner Hospital, Nedlands, WA, 6009, Australia; Medical School, University of Western Australia, Crawley, WA, 6009, Australia; Department of Twin Research & Genetic Epidemiology, King's College London, London, SE1 7EH, UK; Department of Endocrinology & Diabetes, Sir Charles Gairdner Hospital, Nedlands, WA, 6009, Australia; Department of Endocrinology & Diabetes, Sir Charles Gairdner Hospital, Nedlands, WA, 6009, Australia; School of Biomedical Sciences, University of Western Australia, Perth, 6009, Australia; Department of Endocrinology & Diabetes, Sir Charles Gairdner Hospital, Nedlands, WA, 6009, Australia; Department of Endocrinology & Diabetes, Sir Charles Gairdner Hospital, Nedlands, WA, 6009, Australia; Medical School, University of Western Australia, Crawley, WA, 6009, Australia; Population Health Sciences, University of Leicester, Leicester, LE1 7RH, UK; Department of Endocrinology and Metabolism, Odense University Hospital, Odense, 5000, Denmark; Department of Endocrinology and Metabolism, Odense University Hospital, Odense, 5000, Denmark; Department of Endocrinology & Diabetes, Sir Charles Gairdner Hospital, Nedlands, WA, 6009, Australia; Department of Twin Research & Genetic Epidemiology, King's College London, London, SE1 7EH, UK; School of Biomedical Sciences, University of Western Australia, Perth, 6009, Australia; Department of Twin Research & Genetic Epidemiology, King's College London, London, SE1 7EH, UK; Department of Endocrinology & Diabetes, Sir Charles Gairdner Hospital, Nedlands, WA, 6009, Australia; Medical School, University of Western Australia, Crawley, WA, 6009, Australia

**Keywords:** DNA methylation, Hashimoto disease, Graves disease, epigenome

## Abstract

**Context:**

Autoimmune thyroid disease (AITD) includes Graves disease (GD) and Hashimoto disease (HD), which often run in the same family. AITD etiology is incompletely understood: Genetic factors may account for up to 75% of phenotypic variance, whereas epigenetic effects (including DNA methylation [DNAm]) may contribute to the remaining variance (eg, why some individuals develop GD and others HD).

**Objective:**

This work aimed to identify differentially methylated positions (DMPs) and differentially methylated regions (DMRs) comparing GD to HD.

**Methods:**

Whole-blood DNAm was measured across the genome using the Infinium MethylationEPIC array in 32 Australian patients with GD and 30 with HD (discovery cohort) and 32 Danish patients with GD and 32 with HD (replication cohort). Linear mixed models were used to test for differences in quantile-normalized β values of DNAm between GD and HD and data were later meta-analyzed. Comb-p software was used to identify DMRs.

**Results:**

We identified epigenome-wide significant differences (*P* < 9E-8) and replicated (*P* < .05) 2 DMPs between GD and HD (cg06315208 within *MDC1* and cg00049440 within *KLF9*). We identified and replicated a DMR within *CUTA* (5 CpGs at 6p21.32). We also identified 64 DMPs and 137 DMRs in the meta-analysis.

**Conclusion:**

Our study reveals differences in DNAm between GD and HD, which may help explain why some people develop GD and others HD and provide a link to environmental risk factors. Additional research is needed to advance understanding of the role of DNAm in AITD and investigate its prognostic and therapeutic potential.

Autoimmune thyroid disease (AITD) encompasses a spectrum of organ-specific disorders characterized by lymphocytic infiltration of the thyroid gland and production of thyroid autoantibodies. Two of the most common clinical manifestations of AITD are Graves disease (GD) and Hashimoto disease (HD). GD is characterized by stimulatory thyrotropin (TSH) receptor antibodies that result in hyperthyroidism, and HD by a destructive, inflammatory thyroiditis that may progress to hypothyroidism (1, 2). Both conditions are common with a lifetime risk for GD of 3% in women and 0.5% in men, compared with 10% in women and 2% in men for HD (1, 3). Despite the high prevalence of AITD, its pathophysiology remains incompletely understood.

GD and HD are often present in the same family. Genetic studies have identified shared genetic architecture between the 2 conditions, though the estimates range widely between 8% and 55% (4-6). It is unclear why some members of the same family develop GD whereas others develop HD. There are several environmental factors that can affect the risk of AITD; for example, smoking is a risk factor for GD but appears protective in HD, alcohol intake is negatively associated with both GD and HD, iodine supplementation programs increase the incidence of AITD, and a number of infections have been associated with AITD such as hepatitis C, which increases the risk of HD (7, 8). Epigenetics may provide the missing link between genetics and environmental factors and help explain why some individuals develop GD whereas others develop HD.

Epigenetics involves heritable changes that modify gene expression without altering the DNA dinucleotide sequence. DNA methylation (DNAm) is the most widely studied epigenetic mechanism, involving the addition of a methyl group to a cytosine in a cytosine-phosphate-guanine (CpG) dinucleotide sequence, through the activity of DNA methyltransferases (9, 10). DNAm can exert regulatory effects by modulating the binding of regulatory elements to DNA (11). The relationship between DNAm and gene expression is complex; however, typically DNAm in the promoter region of a gene represses gene transcription whereas DNAm in the gene body can increase gene transcription (12). The DNAm profile of individual cell types is unique, thereby facilitating control of gene expression, and is important in regulating the function of specific cell types, including cells involved in the immune system (13). DNAm is affected by a number of environmental factors that are also known to affect the risk of AITD such as smoking, iodine intake, and hepatitis C infection (14).

Research into the role of epigenetics in autoimmune conditions has grown substantially in recent years (15). In the context of autoimmune rheumatic conditions, epigenome-wide association studies have highlighted differences in DNAm associated with the presence of disease, disease activity, and subtype, as well as response to different medications (13, 16). By comparison, however, studies of DNAm in AITD are limited (14). Global DNA hypomethylation was observed in newly diagnosed GD patients that subsequently normalized following therapy, potentially due to changes in disease activity, thyroid hormone levels, or effect of treatment on DNAm (17). An epigenome-wide association study (EWAS) of CD4+ and CD8+ T cells from 38 GD patients and 31 controls reported hypermethylation of the *TSHR* gene and of genes involved in T-cell signaling (18). A limitation was that GD participants were studied approximately 12 years after diagnosis, when disease activity was likely to be minimal. A recent EWAS of pregnant women with HD (13 patient cases, 8 controls) reported differences in DNAm patterns between groups, but the study was not powered to identify individual differentially methylated positions (DMPs) (19).

Since environmental risk factors for AITD are also known to alter DNAm, it is biologically plausible that differential DNAm (particularly in AITD-susceptibility genes and/or immunoregulatory genes) may alter the risk and phenotypic presentation of AITD, potentially explaining why some AITD patients develop GD and others HD. In this study, we performed an epigenome-wide study comparing whole-blood DNAm of patients with GD with HD to identify DMPs and differentially methylated regions (DMRs) between the 2 conditions.

## Materials and Methods

### Study Population

#### Discovery cohort

Patients were recruited as previously described (20). In brief, patients with GD or HD were recruited from outpatient clinics at Sir Charles Gairdner Hospital, a tertiary hospital in Perth, Western Australia. GD was defined as hyperthyroidism with either positive TSH receptor antibodies or diffuse tracer uptake at thyroid scintigraphy. HD was defined as hypothyroidism with increased thyroid peroxidase antibody concentrations or histology demonstrating lymphocytic thyroiditis. Patients with and without goiter were included in both groups. Clinical data including age, sex, age at diagnosis, history of other autoimmune diseases, and treatments to date were recorded. Venous blood samples were obtained at recruitment and stored at −70 °C.

The present study used blood samples from a subset of the original cohort. We selected 32 patients in the GD group treated only with antithyroid medications (no previous radioactive iodine or thyroidectomy), with the shortest time between diagnosis and recruitment and with no history of other autoimmune disease. We selected 32 patients in the HD group who were treated with levothyroxine, had not undergone thyroidectomy, had the shortest time since diagnosis, and without other autoimmune disease.

We calculated that a sample size of 62 patients was able to detect with 80% confidence a 9% change in methylation of more than 80% of individual CpGs assayed at a permutation analysis significance threshold of *P* = 9E-8. We considered these to represent a realistic effect size for clinically relevant disease mechanisms.

#### Replication cohort

The replication cohort consisted of patients with GD or HD attending endocrinology outpatient clinics at Odense University Hospital, Odense, Denmark, as previously described (21). We selected patients for the present study using the same criteria described earlier.

This study was approved by the University of Western Australia Ethics Committee (2020/ET000100).

### DNA Methylation and Quality Control

DNA was extracted from frozen whole-blood samples and DNAm measured using the Infinium MethylationEPIC BeadChip array (Illumina). R package ENmix version 1.22.6 was used to import array data and to perform background correction of raw intensity data (22). The ENmix-normalized βs were then quantile-normalized. Regression on correlated probes normalization was performed to correct for probe-type bias (23). Cross-reactive and polymorphic probes were removed (24-26). Probes with a detection *P* value of less than 1E-6 were removed and CpGs with missingness (defined as a *P* value <1E-6) in more than 5% of samples were entirely removed from the data set. Sex mismatches were assessed using medial detected fluorescence for probes on the X and Y chromosomes. Samples with a medial probe fluorescence intensity more than 3 SDs below the mean were removed. Principal components analysis was performed to assess the effect of batch and biological effects using PCAtools version 1.2 (27). The EpiSmokEr package was used to predict the smoking status of patients (28). White cell–type composition was estimated using the minfi version 1.32, with reference to the Flow.Sorted.Blood.EPIC data set (29, 30). R version 3.6 was used to perform these analyses.

### Statistical Analysis

#### Epigenome-wide association study of discovery cohort

Linear models were used to test for associations between quantile normalized β values of DNAm in GD and HD, adjusting for age, age squared, sex, predicted white blood cell composition (CD8 T cells, CD4 T cells, natural killer cells, B cells, monocytes, and granulocytes), mean intensity and predicted smoking. Analyses were performed using R version 4.2.1 (including packages lmerTest, data.table, and parallel). The epigenome-wide significant threshold was defined as a *P* value below 9E-8, calculated using a permutation approach that accounts for correlation between CpGs (31). R package EasyStrata was used to create a Miami plot (32).

#### Epigenome-wide association study of replication cohort

Epigenome-wide significant CpGs were investigated in the replication cohort using the same method as described earlier. Replication was achieved if the *P* value was less than .05 and the association was directionally consistent.

#### Epigenome-wide association study meta-analysis

Results from both cohorts were meta-analyzed using METAL software (33). The epigenome-wide significant threshold was defined as a *P* value below 9E-8 (31). For the purposes of graphical representation, we subdivided the significant DMPs into those with genetic, epigenetic, or gene expression associations or known biological roles associated with the following groups: “autoimmune thyroid disease” (which included thyroid eye disease [TED]), “thyroid hormone” levels, and “immune” (including nonthyroidal autoimmune diseases and role in immunology). SRplot was used to illustrate epigenome-wide significant findings (34).

#### Differentially methylated regions

To identify DMRs, we used the results of the EWAS of the discovery cohort and replication cohorts, respectively. Comb-*P* software was used with an analysis window of 300 base pairs, an autocorrelation lag of 300 bases, a seed *P* value of .001, and a minimum of 3 probes per DMR that were directionally consistent. *P* values were reported after Sidak multiple testing correction (*P(cor)*). DMRs reported had a *P(cor)* less than .05. R package CoMET was used to create regional association plots (35).

#### Differential methylated regions meta-analysis

Directionally consistent results from the EWAS meta-analysis were used to identify DMRs. Comb-*P* software was used as described earlier.

#### Further analysis of significant differentially methylated positions

We used GoDMC to assess whether differential DNAm at DMPs replicated in the replication cohort are affected by genetic variants (36).

## Results

### Baseline Characteristics

#### Discovery cohort

Two patient samples were excluded during quality control. Thirty-two patients in the GD group and 30 patients in the HD group were included in the discovery study. Most patients were female (94% in GD group and 90% in HD group) and the median time from diagnosis to recruitment was 0.8 years in the GD group and 2.6 years in the HD group (Table 1). Seven participants in the GD group had TED.

**Table 1.** Characteristics of study patients

	Discovery cohort		Replication cohort	
	GD (n = 32)	HD (n = 30)	GD (n = 32)	HD (n = 32)
Age mean ± SD, y	45.5 ± 13.8	42.9 ± 14.3	40.1 ± 12.6	38.4 ± 13.8
Sex, female (%)	30 (94%)	27 (90%)	29 (91%)	29 (91%)
Smoking*^a^*				
Current smoker	8	6	9	5
Former smoker	4	0	0	0
Never smoker	20	24	23	27
Time since diagnosis, y, median (Q1-Q3)	0.8 (0.3-1.0)	2.6 (1.0-4.4)	1 (1-1)	1 (1-1)

Abbreviations: GD, Graves disease; HD, Hashimoto disease; Q1, first quartile; Q3, third quartile.

^*a*^Smoking status was imputed using EpiSmokEr software.

#### Replication cohort

The replication cohort included 32 patients in the GD group and 32 in the HD group; most patients were female (91% in both groups) and median time from diagnosis was 1 year in both groups (see Table 1). One participant in the GD group had TED.

### Epigenome-wide Association Study

The EWAS of the discovery cohort identified 6 DMPs between GD and HD (Table 2, Supplementary Fig. S1 (37)), 2 of which (cg00049440 within *KLF9* and cg06315208 within *MDC1*) were confirmed in the replication cohort. A further 2 DMPs (cg13843840 within *ZMIZ1* and cg03633435 within *HORMAD2-AS1*) were of borderline significance in the replication cohort but were significant in the meta-analysis.

**Table 2.** Epigenome-wide significant differentially methylated positions from the discovery cohort

CpG	Chr	Position (hg19)	Nearest gene	Location	Discovery cohort estimate	Discovery cohort *P*	Replication cohort estimate	Replication cohort *P*	Meta-analysis estimate	Meta-analysis *P*
cg11221509	1	220 684 912	*MARK1*	Intergenic	−1.09	8.88E-8	−0.19	.44	−0.76	2.20E-7
cg06315208	6	30 684 407	*MDC1*	5′ UTR	−0.97	4.38E-8	−0.55	.0069	−0.80	8.13E-11
cg00049440	9	73 026 643	*KLF9*	Body	1.30	2.40E-8	0.62	.010	0.99	1.34E-10
cg13843840	10	80 963 128	*ZMIZ1*	Body	−1.15	3.56E-8	−0.38	.054	−0.78	7.39E-9
cg27064684	15	94 614 694	*LINC01581*	Body	−1.14	6.45E-8	−0.23	.161	−0.62	6.27E-7
cg03633435	22	30 440 436	*HORMAD2-AS1*	Body	−1.02	4.05E-8	−0.35	.082	−0.74	6.67E-9

Abbreviations: Chr, chromosome; CpG, cytosine-phosphate-guanine.

### Epigenome-wide Association Study Meta-Analysis

**Figure 1.**
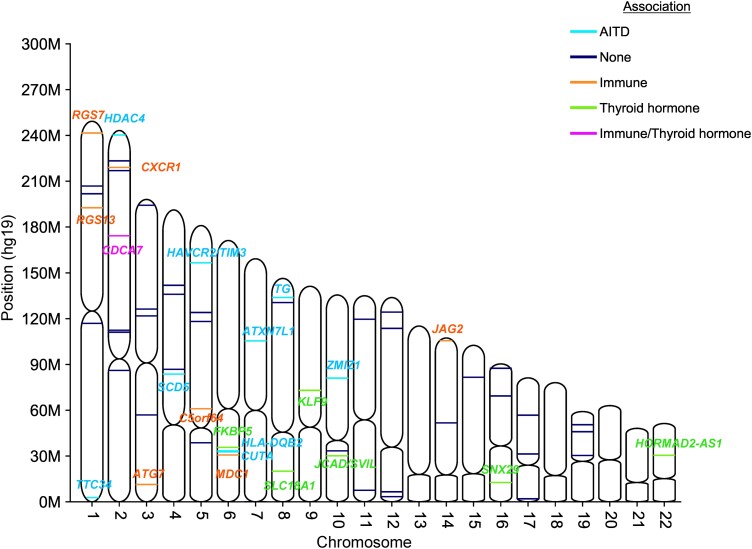
Genomic positions of epigenome-wide significant differentially methylated positions in meta-analysis and their known associations. AITD, autoimmune thyroid disease.

The meta-analysis of discovery and replication cohorts identified 64 DMPs between GD and HD (Supplementary Table S1 (37)). Twelve DMPs were within, or closest to, genes previously reported associated with AITD: A total of 10 DMPs were identified as relevant to immune system function or associated with nonthyroidal autoimmune diseases, and 7 had previously reported associations with thyroid hormone levels (Fig. 1).

### Effect of Genetic Variation on Significant Differentially Methylated Positions

A review of the data in GoDMC identified numerous independent cisgenetic variants that affect DNAm at cg06315208 within *MDC1* and both cisgenetic and transgenetic variants at cg00049440 within *KLF9* (Supplementary Table S2 and Supplementary Table S3, respectively (37)). All transgenetic variants of cg00049440 were located within *THRB*, which encodes a nuclear hormone receptor for triiodothyronine. To our knowledge, none of the identified genetic variants have been detected in previous genome-wide association studies (GWAS) of AITD.

### Differentially Methylated Regions

**Figure 2.**
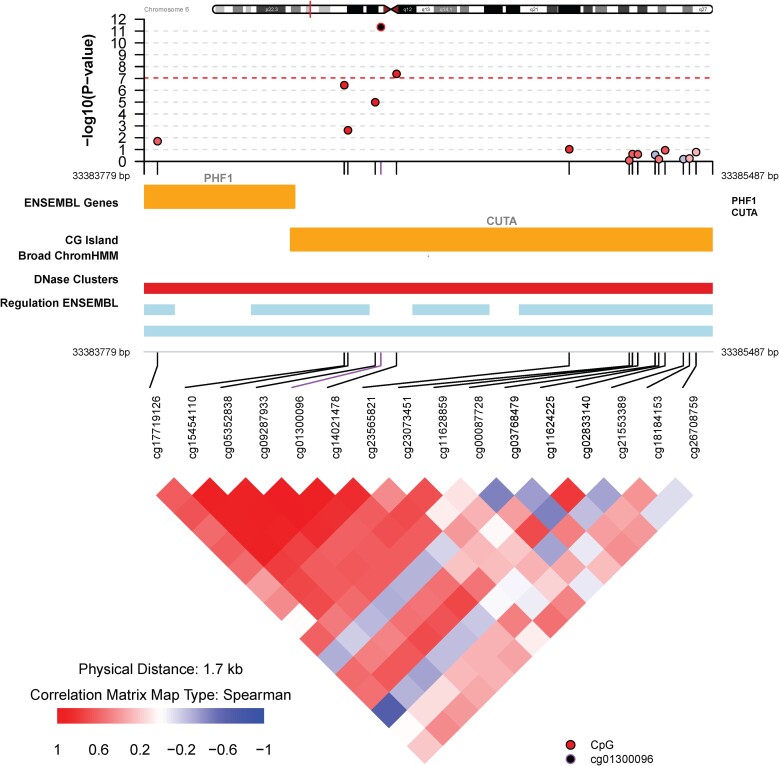
Local association plot showing the genomic region for the differentially methylated region within *CUTA* using results from the meta-analysis (top panel), the functional annotation (middle panel), and the pattern of comethylation.

We identified 29 DMRs between GD and HD in the discovery cohort (Supplementary Table S4 (37)). We replicated a DMR containing 5 probes within *CUTA* (hg19 chromosome 6:33384380-33384537) with an unadjusted *P* value 2.25E-12 (*P(cor)* 1.11E-8) in the discovery cohort and unadjusted *P* value 4.73E-9 (*P(cor)* 1.71E-5) in the replication cohort (Fig. 2).

### Differentially Methylated Regions of Meta-analysis

Our meta-analysis revealed 137 DMRs between GD and HD (Supplementary Table S5 (37)). The top 5 DMRs were within *CUTA*, *ZMIZ1*, *ABI3*, *TG*, and closest to *PFKFB3*.

## Discussion

In this EWAS, we demonstrated differential DNAm between GD and HD. Specifically, we identified and replicated 2 novel DMPs between GD and HD (in *KLF9* and *MDC1*), provide suggestive evidence for a further 2 DMPs (in *ZMIZ1* and *HORMAD2-AS1*) and identified and replicated a region within *CUTA* that was differentially methylated between GD and HD.

Our study showed higher DNAm of cg00049440 within *KLF9* in HD compared to GD. Differential methylation of this CpG has been demonstrated in association with circulating thyroid hormone levels (free 3,5,3′-triiodothyronine, free thyroxine [fT4], and TSH (38, 39)). *KLF9* is demethylated in response to thyroid hormone, and the differential methylation identified in our study is likely a reflection of hyperthyroidism and hypothyroidism in the GD and HD groups, respectively (40). Reduced DNAm of a DMR within *HORMAD2-AS1* was associated with circulating fT4 levels in a previous EWAS, and in our study, we identified reduced DNAm in cg03633435 within *HORMAD2-AS1* in HD compared to GD in the discovery cohort and meta-analysis, again likely related to thyroid hormone levels (38). Our results corroborate the findings from previous EWAS of thyroid function demonstrating differential DNAm (38, 39). Thyroid hormone levels were not measured at the same time as sample collection for DNAm studies, and the median time from diagnosis to recruitment was 0.8 years and 2.6 years in the GD and HD discovery groups, respectively, and 1 year in both GD and HD replication groups. In the interim, patients had been treated with antithyroid medication or thyroxine for GD and HD respectively, and it is likely that they were euthyroid (or nearly so) by the time of recruitment, yet differential DNAm was still present between the two groups. Further research to investigate the time course of altered DNAm in response to changes in thyroid hormone levels may be of clinical interest and may provide an intracellular marker of thyroid hormone action.

We also demonstrated reduced DNAm of cg06315208 in HD compared to GD. This DMP is within mediator of DNA damage checkpoint 1 (*MDC1*). *MDC1* is within the major histocompatibility complex (MHC) region in chromosome 6, a region known to be important in immune regulation containing many genetic variants associated with autoimmunity, including AITD (41). MDC1 is an important DNA damage response protein (42). During T and B lymphocyte development, variable (V), diversity (D), and joining (J) gene segment recombination (VDJ recombination) occurs to create diverse B- and T-cell receptors and immunoglobulins. This process relies on double stranded DNA breaks and DNA damage response pathway (42). MDC1 is part of this process, although its role is incompletely understood (43). In addition, a single-nucleotide variation (SNV, formerly known as single-nucleotide polymorphism) in *MDC1* has shown a suggestive association through GWAS with immunoglobulin G glycosylation (44). Immunoglobulin glycosylation may alter antibodies to become autoreactive, resulting in autoimmune diseases (45). *MDC1* has not previously been associated with AITD, but given its role in VDJ recombination and potential association with immunoglobulin G glycosylation, it is plausible that it may be involved in the pathophysiology of AITD. It is also conceivable that one of the identified genetic variants may be mediating its effect on AITD risk by altering DNAm at cg06315208, though none of these SNVs have been previously associated with AITD in GWAS.

Reduced DNAm at cg13843840 within *ZMIZ1* in the HD group compared to the GD group was seen in the discovery cohort and was directionally consistent and of borderline significance in the replication cohort. This DMP was significant in the meta-analysis, and a DMR was identified within this gene in the meta-analysis. ZMIZ1 is a coactivator of several signaling pathways including NOTCH1 during T-cell development and transforming growth factor β signaling (46-48). Genetic variants in this gene have been associated with several autoimmune diseases including type 1 diabetes, celiac disease, inflammatory bowel disease, vitiligo, psoriasis, and multiple sclerosis (47-52). Recently, *ZMIZ1* was identified as being significantly upregulated in a transcriptome-wide association study of AITD, increasing its appeal for further research in AITD and its role in autoimmunity (53).

A statistically significant DMR in *CUTA* was observed between GD and HD in the discovery and replication cohorts. *CUTA*, or cutA divalent cation tolerance homologue, is expressed in all tissues; however, its role in humans remains unclear. In the brain, CUTA is involved indirectly with processing of β amyloid precursor protein (54). It has no known role in autoimmune disease, but differential expression of *CUTA* in people with rheumatoid arthritis has been described (55). SNVs in *CUTA* have been associated with thyroid hormone use, of which hypothyroidism was the main indication (56). Recently, a study in people with AITD demonstrated that *CUTA* showed pleiotropic associations with AITD, lending further support to our findings (53).

The meta-analysis highlighted 64 DMPs and 137 DMRs between GD and HD, many with known associations with AITD or immune system function. Two DMPs identified in the meta-analysis (cg03605208 and cg01890120) are closest to, and within, thyroglobulin (*TG*), respectively, and one of the top DMRs within the meta-analysis is within *TG*, a gene known to be involved in AITD (57). Other top DMRs were within or closest to *PFKFB3*, *ARID5B*, and *CUTA*, with a study identifying genetic variants in these genes and *TG* associated with thyroid hormone use (56). rs6479778 within *ARID5B* has also been associated with both GD and HD (58). The results of the meta-analysis further support our findings of differential DNAm between GD and HD although further research is required to replicate the findings in these regions.

Strengths of this study include the use of a well-phenotyped group of patients with AITD. We used samples taken from patients relatively soon after diagnosis, and therefore more likely to have active AITD. We used a well-characterized, high-coverage epigenome-wide platform to measure DNAm. This study also has limitations. First, we did not have thyroid function measures at the time of recruitment, which may have been a confounder. However, access to the data of previous EWAS of thyroid function markers is available to help guide which DMPs and DMRs are related to thyroid function differences (38, 39). Second, this was an association study and therefore causality cannot be established. DNAm is susceptible to reverse causation and confounding by genetics. We identified several genetic variants associated with the DMPs we replicated, though none of these have been identified in AITD GWAS previously. Third, data regarding some clinical parameters such as thyroid volume and thyroid peroxidase antibodies were not available and may be a source of heterogeneity within the HD and GD groups. Although we excluded participants with other autoimmune diseases at the time of recruitment, we cannot know whether they may be susceptible to developing autoimmune disease in the future. Furthermore, this study was not powered to consider the potential role of other drugs (unrelated to thyroid physiology) and detrimental lifestyle factors that may contribute to changes in epigenetics.

Further research to replicate and extend these findings may provide additional insights into the pathophysiology of AITD and may identify potential therapeutic targets. It is critical that further studies in epigenetics ensure participants are carefully phenotyped, if they are to deliver valid and informative results.

In conclusion, we have identified altered DNAm that differs between GD and HD. This may be part of the mechanism whereby some people develop GD whereas others develop HD, adding to the complexity of the etiology of AITD (59). Further research to explore the role of differential methylation in AITD is clearly warranted, including DNAm as a potential biomarker in AITD and potential future therapeutic targets. Functional studies looking at the role of *MDC1* and *CUTA* in AITD should also be considered.

## Data Availability

The data sets generated and analyzed during this study are not publicly available but may be accessed through the corresponding author on reasonable request.

## Abbreviations

AITD: autoimmune thyroid disease
CpG: cytosine-phosphate-guanine
*CUTA*: cutA divalent cation tolerance homologue
DMP: differentially methylated position
DMR: differentially methylated region
DNAm: DNA methylation
EWAS: epigenome-wide association study
fT4: free thyroxine
GD: Graves disease
GWAS: genome-wide association study
HD: Hashimoto disease
*MDC1*: mediator of DNA damage checkpoint 1
SNV: single-nucleotide variation
TED: thyroid eye disease
*TG*: thyroglobulin
TSH: thyrotropin
VDJ: variable, diversity, and joining

## References

[1] Smith TJ , Hegedüs L. Graves’ disease. N Engl J Med. 2016;375(16):1552‐1565. 27797318 10.1056/NEJMra1510030

[2] Ralli M , Angeletti D, Fiore M, et al Hashimoto's thyroiditis: an update on pathogenic mechanisms, diagnostic protocols, therapeutic strategies, and potential malignant transformation. Autoimmun Rev. 2020;19(10):102649. 32805423 10.1016/j.autrev.2020.102649

[3] Hiromatsu Y , Satoh H, Amino N. Hashimoto's thyroiditis: history and future outlook. Hormones (Athens). 2013;12(1):12‐18. 23624127 10.1007/BF03401282

[4] Simmonds MJ . GWAS In autoimmune thyroid disease: redefining our understanding of pathogenesis. Nat Rev Endocrinol. 2013;9(5):277‐287. 23529038 10.1038/nrendo.2013.56

[5] Skov J , Calissendorff J, Eriksson D, et al Limited genetic overlap between overt Hashimoto's Thyroiditis and graves’ disease in twins: a population-based study. J Clin Endocrinol Metab. 2020;106(4):1101‐1110. 10.1210/clinem/dgaa956 PMC7993582 33382429

[6] Brix TH , Hegedus L. Twin studies as a model for exploring the aetiology of autoimmune thyroid disease. Clin Endocrinol (Oxf). 2012;76(4):457‐464. 22168537 10.1111/j.1365-2265.2011.04318.x

[7] Effraimidis G , Wiersinga WM. Mechanisms in endocrinology: autoimmune thyroid disease: old and new players. Eur J Endocrinol. 2014;170(6):R241‐R252. 24609834 10.1530/EJE-14-0047

[8] Uldall Torp NM , Liew Z, Carlé A, et al Hyperthyroidism in Danish pregnant women during a 20-year period. J Clin Endocrinol Metab. 2024;109 (1):e370‐e378. 10.1210/clinem/dgad410 37437100

[9] Flanagan JM . Epigenome-wide association studies (EWAS): past, present, and future. Methods Mol Biol. 2015;1238:51‐63. 25421654 10.1007/978-1-4939-1804-1_3

[10] Han L , Zhang H, Kaushal A, et al Changes in DNA methylation from pre- to post-adolescence are associated with pubertal exposures. Clin Epigenetics. 2019;11(1):176. 31791392 10.1186/s13148-019-0780-4 PMC6888960

[11] Jones PA . Functions of DNA methylation: islands, start sites, gene bodies and beyond. Nat Rev Genet. 2012;13(7):484‐492. 22641018 10.1038/nrg3230

[12] Greenberg MVC , Bourc’His D. The diverse roles of DNA methylation in mammalian development and disease. Nat Rev Mol Cell Biol. 2019;20(10):590‐607. 31399642 10.1038/s41580-019-0159-6

[13] De La Calle-Fabregat C , Rodríguez-Ubreva J, Cañete JD, Ballestar E. Designing studies for epigenetic biomarker development in autoimmune rheumatic diseases. Rheumatol Immunol Res. 2022;3(3):103‐110. 36788968 10.2478/rir-2022-0018 PMC9895872

[14] Lafontaine N , Wilson SG, Walsh JP. DNA Methylation in autoimmune thyroid disease. J Clin Endocrinol Metab. 2023;108(3):604‐613. 36420742 10.1210/clinem/dgac664

[15] Wu H , Chen Y, Zhu H, Zhao M, Lu Q. The pathogenic role of dysregulated epigenetic modifications in autoimmune diseases. Front Immunol. 2019;10:2305. 31611879 10.3389/fimmu.2019.02305 PMC6776919

[16] Ballestar E , Sawalha AH, Lu Q. Clinical value of DNA methylation markers in autoimmune rheumatic diseases. Nat Rev Rheumatol. 2020;16(9):514‐524. 32759997 10.1038/s41584-020-0470-9 PMC7955859

[17] Guo Q , Wu D, Yu H, et al Alterations of global DNA methylation and DNA methyltransferase expression in T and B lymphocytes from patients with newly diagnosed autoimmune thyroid diseases after treatment: A follow-up study. Thyroid. 2018;28(3):377‐385. 29336230 10.1089/thy.2017.0301

[18] Limbach M , Saare M, Tserel L, et al Epigenetic profiling in CD4 + and CD8+ T cells from Graves’ disease patients reveals changes in genes associated with T cell receptor signaling. J Autoimmun. 2016;67:46‐56. 26459776 10.1016/j.jaut.2015.09.006

[19] Wenqian C , Fan W, Hu X. Genome-wide DNA methylation analysis of Hashimoto's Thyroiditis during pregnancy. FEBS Open Bio. 2020;10(12):2780‐2790. 10.1002/2211-5463.13018 PMC7714066 33113271

[20] Walsh JP , Berry J, Liu S, et al The clinical presentation of autoimmune thyroid disease in men is associated with IL12B genotype. Clin Endocrinol (Oxf*)*. 2011; 74(4):508‐512. 21198744 10.1111/j.1365-2265.2010.03970.x

[21] Campbell P , Brix TH, Wilson SG , et al Common genetic variants associated with thyroid function may be risk alleles for Hashimoto's Disease and graves’ disease. Clin Endocrinol (Oxf*)*. 2016; 84(2):278‐283. 25683181 10.1111/cen.12746

[22] Xu Z , Niu L, Li L, Taylor JA. ENmix: a novel background correction method for illumina HumanMethylation450 BeadChip. Nucleic Acids Res. 2016;44(3):e20. 26384415 10.1093/nar/gkv907 PMC4756845

[23] Niu L , Xu Z, Taylor JA. RCP: a novel probe design bias correction method for illumina methylation BeadChip. Bioinformatics. 2016;32(17):2659‐2663. 27153672 10.1093/bioinformatics/btw285 PMC5013906

[24] Pidsley R , Zotenko E, Peters TJ, et al Critical evaluation of the illumina MethylationEPIC BeadChip microarray for whole-genome DNA methylation profiling. Genome Biol. 2016;17(1):208. 27717381 10.1186/s13059-016-1066-1 PMC5055731

[25] Chen Y-A , Lemire M, Choufani S, et al Discovery of cross-reactive probes and polymorphic CpGs in the illumina infinium HumanMethylation450 microarray. Epigenetics. 2013;8(2):203‐209. 23314698 10.4161/epi.23470 PMC3592906

[26] Selim S . Illumina 450k Filtering. GitHub Repository. 2018.

[27] Blighe K , Lun A. PCAtools: everything Principal Component Analysis. R package version 1.2.0. R package; 2019.

[28] Bollepalli S , Korhonen T, Kaprio J, Anders S, Ollikainen M. Epismoker: a robust classifier to determine smoking status from DNA methylation data. Epigenomics. 2019;11(13):1469‐1486. 31466478 10.2217/epi-2019-0206

[29] Aryee MJ , Jaffe AE, Corrada-Bravo H, et al Minfi: a flexible and comprehensive bioconductor package for the analysis of infinium DNA methylation microarrays. Bioinformatics. 2014;30(10):1363‐1369. 24478339 10.1093/bioinformatics/btu049 PMC4016708

[30] Salas LA , Koestler DC. FlowSorted.Blood.EPIC: Illumina EPIC Data on Immunomagnetic Sorted Peripheral Adult Blood Cells. R Package Version 1.4.1. R package; 2019.

[31] Mansell G , Gorrie-Stone TJ, Bao Y, et al Guidance for DNA methylation studies: statistical insights from the Illumina EPIC array. BMC Genom. 2019;20(1):366. 10.1186/s12864-019-5761-7 PMC6518823 31088362

[32] Winkler TW , Kutalik Z, Gorski M, Lottaz C, Kronenberg F, Heid IM. Easystrata: evaluation and visualization of stratified genome-wide association meta-analysis data. Bioinformatics. 2015;31(2):259‐261. 25260699 10.1093/bioinformatics/btu621 PMC4287944

[33] Willer CJ , Li Y, Abecasis GR. METAL: fast and efficient meta-analysis of genomewide association scans. Bioinformatics. 2010;26(17):2190‐2191. 20616382 10.1093/bioinformatics/btq340 PMC2922887

[34] Tang D , Chen M, Huang X, et al SRplot: A free online platform for data visualization and graphing. PLoS One. 2023;18(11):e0294236. doi:10.1371/journal.pone.0294236 37943830 PMC10635526

[35] Martin TC , Yet I, Tsai P-C, Bell JT. coMET: visualisation of regional epigenome-wide association scan results and DNA co-methylation patterns. BMC Bioinform. 2015;16(1):131. 10.1186/s12859-015-0568-2 PMC4422463 25928765

[36] Genetics of DNA Methylation Consortium. Vol 2023.

[37] Lafontaine Bedecarratz N . Data from: Supplement for: Epigenome-Wide Association Study Shows Differential DNA Methylation of MDC1, KLF9 and CUTA in Autoimmune Thyroid Disease. The University of Western 2023. Deposited August 3, 2023. doi:10.26182/et7h-6k03. PMC10940258 37962983

[38] Lafontaine N , Campbell PJ, Castillo-Fernandez JE, et al Epigenome-Wide association study of thyroid function traits identifies novel associations of fT3 with KLF9 and DOT1L. J Clin Endocrinol Metab. 2021;106(5):e2191‐e2202. 33484127 10.1210/clinem/dgaa975 PMC8063248

[39] Weihs A , Chaker L, Martin TC, et al Epigenome-wide association study reveals CpG sites associated with thyroid function and regulatory effects on KLF9. Thyroid. 2023;33(3):301‐311. 36719767 10.1089/thy.2022.0373 PMC10024591

[40] Raj S , Kyono Y, Sifuentes CJ, Arellanes-Licea EDC, Subramani A, Denver RJ. Thyroid hormone induces DNA demethylation in Xenopus tadpole brain. Endocrinology. 2020;161(11):bqaa155. 32865566 10.1210/endocr/bqaa155 PMC7947600

[41] Giuliani C , Verrocchio S, Verginelli F, Bucci I, Grassadonia A, Napolitano G. Hormonal regulation of the MHC class I gene in thyroid cells: role of the promoter “tissue-specific” region. Front Endocrinol (Lausanne). 2021;12:749609. 34938270 10.3389/fendo.2021.749609 PMC8685237

[42] Salguero I , Belotserkovskaya R, Coates J, et al MDC1 PST-repeat region promotes histone H2AX-independent chromatin association and DNA damage tolerance. Nat Commun. 2019;10(1):5191. 31729360 10.1038/s41467-019-12929-5 PMC6858307

[43] Beck C , Castañeda-Zegarra S, Huse C, Xing M, Oksenych V. Mediator of DNA damage checkpoint protein 1 facilitates V(D)J recombination in cells lacking DNA repair factor XLF. Biomolecules. 2019;10(1):60. 31905950 10.3390/biom10010060 PMC7023129

[44] Lauc G , Huffman JE, Pučić M, et al Loci associated with N-glycosylation of human immunoglobulin G show pleiotropy with autoimmune diseases and haematological cancers. PLoS Genet. 2013;9(1):e1003225. 23382691 10.1371/journal.pgen.1003225 PMC3561084

[45] Zhou X , Motta F, Selmi C, Ridgway WM, Gershwin ME, Zhang W. Antibody glycosylation in autoimmune diseases. Autoimmun Rev. 2021;20(5):102804. 33727152 10.1016/j.autrev.2021.102804 PMC8058319

[46] Trynka G , Wijmenga C, van Heel DA. A genetic perspective on coeliac disease. Trends Mol Med. 2010;16(11):537‐550. 20947431 10.1016/j.molmed.2010.09.003

[47] Imielinski M , Baldassano RN, Griffiths A, et al Common variants at five new loci associated with early-onset inflammatory bowel disease. Nat Genet. 2009;41(12):1335‐1340. 19915574 10.1038/ng.489 PMC3267927

[48] Lomelí H . ZMIZ Proteins: partners in transcriptional regulation and risk factors for human disease. J Mol Med (Berl). 2022;100(7):973‐983. 35670836 10.1007/s00109-022-02216-0

[49] Wang N , Shen N, Vyse TJ, et al Selective IgA deficiency in autoimmune diseases. Mol Med. 2011;17(11-12):1383‐1396. 21826374 10.2119/molmed.2011.00195 PMC3321806

[50] Sun Y , Zuo X, Zheng X, et al A comprehensive association analysis confirms ZMIZ1 to be a susceptibility gene for vitiligo in Chinese population. J Med Genet. 2014;51(5):345‐353. 24667117 10.1136/jmedgenet-2013-102233

[51] Sawcer S , Hellenthal G, Pirinen M, et al Genetic risk and a primary role for cell-mediated immune mechanisms in multiple sclerosis. Nature. 2011;476(7359):214‐219. 21833088 10.1038/nature10251 PMC3182531

[52] Nishimura A , Matsumura K, Kikuno S, et al Slowly progressive type 1 diabetes Mellitus: current knowledge and future perspectives. Diabetes Metab Syndr Obes. 2019;12:2461‐2477. 31819572 10.2147/DMSO.S191007 PMC6886592

[53] Liu X , Miao Y, Liu C, Lu W, Feng Q, Zhang Q. Identification of multiple novel susceptibility genes associated with autoimmune thyroid disease. Front Immunol. 2023;14:1161311. 37197658 10.3389/fimmu.2023.1161311 PMC10183592

[54] Zhao Y , Wang Y, Hu J, Zhang X, Zhang Y-W. Cuta divalent cation tolerance homolog (Escherichia coli) (CUTA) regulates β-cleavage of β-amyloid precursor protein (APP) through interacting with β-site APP cleaving protein 1 (BACE1). J Biol Chem. 2012;287(14):11141‐11150. 22351782 10.1074/jbc.M111.330209 PMC3322838

[55] Zhu H , Xia W, Mo X-B, et al Gene-Based genome-wide association analysis in European and Asian populations identified novel genes for rheumatoid arthritis. PLoS One. 2016;11(11):e0167212. 27898717 10.1371/journal.pone.0167212 PMC5127563

[56] Wu Y , Byrne EM, Zheng Z, et al Genome-wide association study of medication-use and associated disease in the UK Biobank. Nat Commun. 2019;10(1):1891. 31015401 10.1038/s41467-019-09572-5 PMC6478889

[57] Vargas-Uricoechea H . Molecular mechanisms in autoimmune thyroid disease. Cells. 2023;12(6):918. 36980259 10.3390/cells12060918 PMC10047067

[58] Tomer Y , Hasham A, Davies TF, et al Fine mapping of loci linked to autoimmune thyroid disease identifies novel susceptibility genes. J Clin Endocrinol Metab. 2013;98(1):E144-152. 23118423 10.1210/jc.2012-2408 PMC3537111

[59] Brix TH , Hegedüs L. The complexity of the etiology of autoimmune thyroid disease is gravely underestimated. Thyroid. 2011;21(12):1289‐1292. 22136263 10.1089/thy.2011.2112.ed

